# The Impact of Baseline Pain Intensity on the Effectiveness of Whole-Body Electromyostimulation (WB-EMS) for Nonspecific Chronic Back Pain

**DOI:** 10.7759/cureus.57858

**Published:** 2024-04-08

**Authors:** Karl L Konrad, Anja Weissenfels, Christof Birkenmaier, Jean-Pierre Baeyens, Wolfgang Kemmler, Bernd Wegener

**Affiliations:** 1 Department of Orthopaedic Surgery, Physical Medicine and Rehabilitation, Ludwig Maximilian University (LMU), Munich, DEU; 2 Experimental Anatomy (EXAN) Research Group, Department of Physiotherapy, Human Physiology and Anatomy, Vrije Universiteit Brussel (VUB), Brussel, BEL; 3 Therapy Research, Institut Ascend, Röfingen, DEU; 4 Institute of Medical Physics, Friedrich-Alexander University, Erlangen, DEU; 5 Department of Orthopaedics, Artemed Klinikum München Süd, Munich, DEU; 6 Department of Research, THIM University College Physiotherapy, Landquart, CHE

**Keywords:** whole-body electromyostimulation (wb-ems), nonspecific chronic back pain (nscbp), pain measurement, neuromuscular electrical stimulation (nmes), electrotherapy, pain intensity, exercise therapy, electric stimulation therapy

## Abstract

Introduction: Recent clinical studies confirmed that whole-body electromyostimulation (WB-EMS) training is a safe and time-efficient therapeutic method for patients with nonspecific chronic back pain (NSCBP). However, significant variations in initial pain intensity among subjects in these studies have been observed. This study aims to determine if patients with differing initial pain intensities experience varying degrees of benefit from WB-EMS and to assess the overall correlation between initial pain levels and pain reduction.

Methods: Pain intensity datasets from two studies were combined. The pooled data included 121 NSCBP patients (38 males and 83 females) with an average age of 55.1 years (±11.8 years). Data was categorized by baseline pain intensity on the numeric rating scale (NRS) into seven groups: 0 to 2, >2 to 3, >3 to 4, >4 to 5, >5 to 6, >6 to 7, and >7. Both absolute and relative changes were analyzed. Additionally, a Spearman rho correlation test was performed on the entire dataset to evaluate the relationship between initial pain level and pain reduction.

Results: Significant improvements were noted across all NRS11 categories, with strong effect sizes (p) in all classes above 2, ranging from 0.56 to 0.90. The >7 category exhibited the highest rate of clinically significant changes (80%) and an average improvement of 3.72 points. The overall group from >1 to 10 showed an average improvement of 1.33 points, with 37% of the participants experiencing clinically significant improvements. The Spearman rho correlation test revealed a moderate positive relationship between initial pain level and pain reduction (r_s = 0.531, p < 0.001), indicating that, generally, higher initial pain levels are associated with greater pain reduction.

Conclusion: The findings support the hypothesis that NSCBP patients with higher baseline NRS values benefit more substantially from WB-EMS. Those with NRS values above 7 show the greatest improvement and highest rate of clinical significance. The overall positive correlation between initial pain intensity and pain reduction further underscores the efficacy of WB-EMS in managing NSCBP across different pain intensities.

## Introduction

Nonspecific chronic back pain (NSCBP) is a predominant cause of disability worldwide, significantly impacting the quality of life and leading to an increase in disability-adjusted life years (DALYs) [1]. It is the leading chronic disease that forces more people out of their jobs than diabetes, heart disease, hypertension, respiratory diseases, asthma, and neoplasm combined [2,3]. While predominantly affecting individuals aged between 40 and 69, NSCBP does not discriminate, as it can occur at any age and is more frequently diagnosed in females [4]. The prevalence of NSCBP is notably higher in high-income countries, suggesting a correlation with sedentary lifestyles facilitated by a robust human development index [5]. With approximately 90% of cases lacking a specific anatomical correlation [6], the term "nonspecific" underscores the enigmatic nature of this condition's etiology, which ranges from one-sided movements and repetitive strain to overall physical inactivity [7-9], which may lead to a reduction of muscle power and strength, particularly in the trunk [10].

Despite the burgeoning evidence supporting active exercise therapy, including the latest recommendations from the World Health Organization, as a promising treatment method for NSCBP [6,11-13], engagement remains low. Barriers such as coexisting conditions and the demands of modern life frequently hinder the initiation and maintenance of a consistent exercise regimen [14]. In this context, whole-body electromyostimulation (WB-EMS) has emerged as an innovative therapeutic modality, offering a joint-friendly and time-efficient alternative that could potentially address the abovementioned main barriers to physical exercise [15]. Research has shown promising results for WB-EMS in improving muscle performance [16-26].

Clinical trials specifically investigating NSCBP patients suggest that WB-EMS training can significantly reduce pain intensity, offering a valuable alternative to traditional exercise therapies.

Weissenfels et al. [27] conducted a clinical trial where 110 patients with low back pain (LBP) aged 40-70 years (the age group with the highest prevalence) were divided into a WB-EMS group (n = 55) and an active control group (ACG) (n = 55). The WB-EMS group reported a mean baseline pain intensity of 2.69 (±1.52) on a numeric rating scale (NRS). This score significantly improved by 0.60 (±0.96) points after 12 weeks of WB-EMS training.

Another study by Konrad et al. [28] involved 128 NSCBP patients, with 85 patients in the WB-EMS group and 43 patients receiving a multimodal back pain approach. Compared to the study of Weissenfels et al., the baseline pain intensity in the WB-EMS group was 1.76 points higher at 4.45 (±2.02). Their pain score improved after 12 weeks with 1.58 (±2.02) points also at a higher degree.

This observation of significantly different improvements with varying baseline NRS11 values led to the following questions: (1) do patients with different pain intensities at baseline benefit from WB-EMS to a different degree and (2) do significant improvements occur at all baseline NRS categories?

Thesis statement

This study is the first that aims to evaluate the differential impact of WB-EMS on patients with different levels of baseline pain intensity, thereby offering comprehensive insights into its efficacy across a broad spectrum of NSCBP severity. By systematically identifying which patient groups stand to benefit the most from WB-EMS, our research endeavors to inform tailored treatment plans that could significantly enhance patient outcomes and overall quality of life in the face of NSCBP.

The imperative to understand the nuanced benefits of WB-EMS across diverse patient profiles is not just an academic exercise but a clinical necessity, poised to redefine NSCBP management strategies. Our findings, presented at the International Society of Physical and Rehabilitation Medicine (ISPRM) 2022 World Congress in Lisbon and the German Congress for Orthopedics and Trauma Surgery 2022, underline the potential of initial pain levels as a predictive marker for therapeutic outcomes, marking a pivotal step toward personalized patient care in NSCBP treatment.

## Materials and methods

This study synthesizes and analyzes data from two controlled intervention studies conducted by Konrad et al. [27] and Weissenfels et al. [28], which examined the impact of whole-body electromyostimulation (WB-EMS) on patients with nonspecific chronic back pain (NSCBP), focusing on pain intensity as the primary outcome measure. Ethical approval for both source studies was obtained from the respective universities' ethics commissions (Munich and Erlangen), and they were conducted in accordance with the Declaration of Helsinki. Furthermore, both trials were registered in the German Clinical Trials Register (Konrad et al.: DRKS00011896, Weissenfels et al.: DRKS00009528), ensuring their inclusion in the International Clinical Trials Registry Platform (ICTRP) of the World Health Organization (WHO).

Data source and pooling

The analysis is based on raw, anonymized participant data underlying the aforementioned studies. Both studies employed similar settings, impulse parameters, and exercises, making the interventions and assessment methods highly comparable. This study's pooled dataset includes 121 NSCBP patients (38 males and 83 females), with an average age of 55.1 years (±11.8 years), stratified by baseline pain intensity on the numeric rating scale (NRS) into categories ranging from 0 to 2, >2 to 3, >3 to 4, >4 to 5, >5 to 6, >6 to 7, and >7 (Table 1).

**Table 1:** Comparison of NRS baseline outcomes in the source studies N = number of subjects, NRS baseline = pain intensity on numeric rating scale at baseline, ∆ 12 weeks = delta after 12 weeks of intervention

Study	N	NRS baseline	∆ 12 weeks
Weissenfels et al. [27]	55	2.69 (±1.52)	-0.60 (±0.96)
Konrad et al. [28]	86	4.45 (±2.2)	-1.58 (±2.02)

Studies by Weissenfels et al.

In September 2018, Weissenfels et al. [29] published data from their project in progress. They reported significantly higher improvements in pain intensity of patients trained with WB-EMS (n = 15) as compared to a passive control group (n = 15). In 2019, the data of 55 NSCBP patients in the WB-EMS group of the abovementioned project were published [27]. The 15 subjects from the first publication [29] are herein also included. Participants were allocated to either a WB-EMS group or a conventional back-strengthening protocol (n = 55) using a stratified randomization method, which involved drawing lots after stratifying them into groups based on pain intensity on the NRS (0-3, 4-7, and 7-10). The WB-EMS group was compared to a conventional back-strengthening protocol (n = 55). Both groups showed comparable (and significant) improvements. Seven of the 55 subjects in the EMS group dropped out. As a result, 48 complete datasets are available for analysis; this includes the EMS datasets from both publications.

Training modalities: The characteristics of the electrical current were a symmetric, bipolar, rectangular impulse with 350 microseconds impulse width and an impulse frequency of 85 Hertz in an interval of six seconds pulse duration and four seconds pulse break. While electrically stimulated, participants performed movements with low intensity: squat with latissimus pulleys, butterfly reverse (with angled arms), straight pullovers with trunk flexion (lumberjacks), standing trunk flexion (crunch), one-legged stand with biceps curl, and sidestep with weight shift and biceps curl. Subjects were asked to exercise at a perceived exertion rate (RPE) between "hard" (5) and "very hard" (7) according to the Borg CR-10 scale [30,31]. The time was increased from 12 to 20 minutes in the first four sessions, with a time increase of two minutes per session. Participants trained once a week.

Assessment and outcome: Pain intensity was assessed with an NRS11 scale from 0 (no pain) to 10 (worst imaginable pain), which was conducted during four weeks before and during the last four weeks of the intervention [32]. Participants were requested to rate their highest daily pain intensity on a standardized questionnaire. The average pain intensity at four weeks before and during the last four weeks of the 12-week lasting intervention was used for the analysis of the primary endpoint.

Study by Konrad et al.

In the study by Konrad et al. [28] (2020), 85 patients with NSCBP participated in the WB-EMS group and 43 in the active control group (ACG). Thirty-four participants without back pain were included as a peer group. In the WB-EMS group, eight participants dropped out, and the NRS data after 12 weeks of four participants was not available. Consequently, datasets for the complete 12-week follow-up of 73 subjects were available for analysis of the pain intensity.

Training modalities: The basic characteristics of the electrical current were the same as those used by Weissenfels et al. (2018 and 2019): symmetric, bipolar, rectangular impulse with 350 microseconds impulse width and an impulse frequency of 85 Hertz. However, the duty cycle in this study was four seconds pulse duration and four seconds pulse break. Using the "back strengthening" program of the WB-EMS device, the subjects performed the following dynamic exercises: squat, butterfly reverse left, butterfly reverse right, trunk rotation left, trunk rotation right, left diagonal crunches, right diagonal crunches, straight crunches, trunk extension, and table position. All exercises were carried out in an upright position, without external load. The training frequency was also once a week for 20 minutes (except the first session, which was six minutes of impulse familiarization and 12 minutes of training). During the first six sessions, the limit for perceived intensity was set to a maximum of "5 strong" on the Borg CR-10 RPE scale [30,31]. From the sixth to the ninth session, the perceived intensity was gradually increased to levels between "7 very strong" and "8."

Assessment and outcome: In this study, the subjects were instructed to cross one of 11 equally sized fields (numbered from 0 to 10) of a horizontal bar with the number representing the actual pain intensity from 0 (no pain) to 10 (worst imaginable pain). These values were validated with other pain outcome variables as the North American Spine Society (NASS) Lumbar Spine Outcome Assessment Instrument and the 36-Item Short Form Survey (SF-36) pain parameters. Assessments were applied at the following times: T0 (baseline): before treatment, T1: after six weeks of treatment, T2: after 12 weeks of treatment, and T3: after six months of treatment (complete training period). Since the observation period in the study by Weissenfels et al. [27] was 12 weeks, the T0 and T2 (also 12 weeks) values of their datasets were used.

Data analysis and stratification

From both source studies (Weissenfels et al. [27] and Konrad et al. [28]), all complete pain intensity datasets (assessed by the NRS) throughout the 12-week follow-up period were pooled in one database. Since the NRS is an ordinal scale variable, nonparametric tests were employed for analysis.

To explore the general influence of initial pain level (NRS) on pain reduction, a Spearman rho correlation test of the whole database was performed.

Stratification was done according to the initial pain intensity (NRS) into the following categories: 0 to 2, >2 to 3, >3 to 4, >4 to 5, >5 to 6, >6 to 7, and all values greater than 7. Furthermore, a category was formed of all subjects who had a pain intensity greater than one (>1 to 10) at baseline. As every change of two or more points on the NRS is stated as clinically relevant [33], absolute and relative frequencies were also analyzed. For easier comparability, the categorization according to the Verbal Descriptor Scale (VDS) [34] is displayed in graphs and tables.

For descriptive statistics, means and standard deviations (SDs) were used for continuous variables, and absolute and relative frequencies for categorical variables. To compare pairwise categorical measurements, the Wilcoxon signed-rank test was performed, since the NRS is an ordinal scale variable. For effect size (ES), the Pearson correlation coefficient (r) was calculated from Z scores (formula: r = |Z/(√n)|). The level of significance was set at p < 0.05 (5%).

As some studies suggest that age and gender may influence the prognosis of chronic pain [35-38], these variables were identified as potential confounders that might influence the prognosis of therapy in nonspecific chronic back pain. Consequently, statistical methods were applied to control for these variables. Specifically, the influence of gender on NRS changes was examined using the Mann-Whitney U test. Furthermore, the impact of age on NRS changes was assessed by a Spearman rho correlation analysis to explore rank-based associations. These statistical approaches were chosen to robustly evaluate the potential effects of age and gender on treatment outcomes, ensuring the validity of our findings.

Analysis was done using Statistical Package for Social Sciences (SPSS) version 22.0 for Windows (IBM SPSS Statistics, Armonk, NY).

## Results

Sample description

The pooled database included 121 complete datasets for pain intensity assessments both at baseline and after a 12-week follow-up period. The participants ranged in age from 26 to 86 years, with a mean age of 55.1 ± 11.8 years, comprising 38 male and 83 female subjects. The mean baseline pain intensity across the cohort was 3.83 on the numeric rating scale (NRS) (Table 2).

**Table 2:** Sample description N = number of subjects, NRS_T0 = pain intensity on numeric rating scale at baseline, NRS_12weeks = pain intensity on numeric rating scale after 12 weeks of intervention, Min = minimal observed value, Max = maximum observed value, SD = standard deviation

Group		N	Min	Max	Mean	SD
Weissenfels et al. [27]	Age	55	40.0	70.0	54.4	7.4
	NRS_T0	55	0.2	7.3	2.7	1.8
	NRS_12weeks	48	0.0	5.3	1.9	1.4
Konrad et al. [28]	Age	85	25.0	86.0	55.7	13.7
	NRS_T0	84	1.0	10.0	4.6	1.8
	NRS_12weeks	73	0.0	7.0	3.0	2.0
Combined cohort (valid)	Age	121	26.0	86.0	55.1	11.8
	NRS_T0	121	0.2	10.0	3.8	2.0
	NRS_12weeks	121	0.0	7.0	2.6	1.8

Potential confounders

In the analysis with a Spearman rho correlation test, age showed a very weak negative correlation with pain reduction (r_s = -0.073, p = 0.447), indicating no significant relationship. Gender differences in pain reduction were analyzed using the Mann-Whitney U test, revealing also no statistically significant difference between males and females in terms of pain reduction (U = 1141.500, p = 0.283), despite females showing a slightly higher mean rank (58.18) compared to males (51.07).

Concerning research question

The comprehensive analysis revealed a moderate positive correlation between initial pain intensity and subsequent pain reduction (Spearman rho r_s = 0.531, p < 0.001), indicating that higher initial pain levels generally correspond to greater reductions in pain.

**Figure 1:**
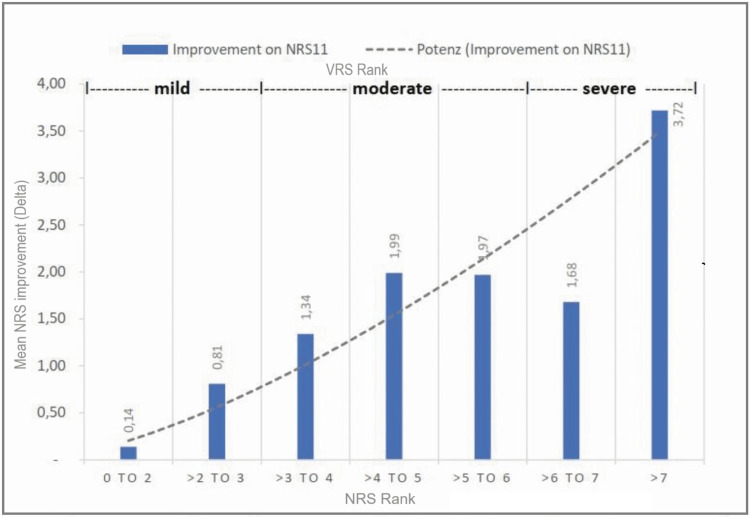
Improvements in NRS11 in the different categories Changes in different classes on the NRS11, with exponential trend line (potenz). Additionally, pain categories mild, moderate, and severe according to the VDS are shown. NRS = numeric rating scale, VDS = Verbal Descriptor Scale

**Figure 2:**
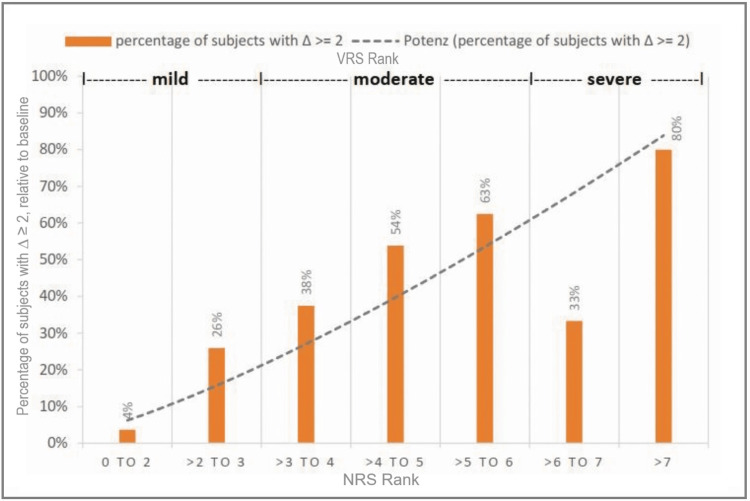
Rate of subjects who show clinically significant improvements (≥2) Percentage of subjects with an improvement of a minimum of 2 points (∆ ≥ 2) on NRS relative to baseline. Additionally, pain categories mild, moderate, and severe according to the VDS are shown. NRS = numeric rating scale, VDS = Verbal Descriptor Scale, potenz = exponential trend line

In addition, all pain intensity categories demonstrated significant improvements. The effect size (r) of the improvements observed in the pain categories with a pain score greater than 2 was between 0.56 and 0.90, which means a strong effect (Table 3). The effect size was weak only in the category "0 to 2" with 0.18 and showed 0.14 the lowest mean improvement and 4% the lowest rate of clinical improvements. The class ">2 to 3" demonstrated a mean improvement of 0.81, and 26% of the subjects improved clinically significantly (>2 on NRS). In the class ">3 to 4," the improvement was 1.34, and 38% of the subjects improved clinically significantly. From ">4 to 5," 1.99 points mean improvement was observed, while 54% of the subjects improved clinically significantly. The class ">5 to 6" showed a mean improvement of 1.97 points, and 63% improved by 2 or more points. In class ">6 to 7," 1.68 points lowering of pain intensity was observed, while 33% of the participants showed clinically significant changes. The class ">7" showed the highest rate of clinically significant changes, being 80%, and improved by a mean of 3.72 from baseline. Without very low values (1 and smaller), the entire group from ">1 to 10" shows a mean improvement of 1.33 points, while 37% of the participants improved 2 or more points (Figure 1 and Figure 2).

**Table 3:** Changes in pain intensity in stratified classes * = statistically significant (p < 0.05), VDS = Verbal Descriptor Scale, NRS = numeric rating scale, n = number of subjects in the subgroup, SD = standard deviation, ∆ = mean change (delta), ∆ rel = change relative to baseline, ES = effect strength (r), ∆ >=2 = absolute numbers and rate of subjects that improved 2 or more points

VDS rank	NRS rank	n	Mean	SD	∆	∆_ rel_	ES	∆ >= 2	
Mild	0 to 2	27	1.32	0.60	-0.14*	-11%	0.18	1	4%
	>2 to 3	27	2.75	0.31	-0.81*	-29%	0.58	7	26%
Moderate	>3 to 4	24	3.85	0.28	-1.34*	-35%	0.67	9	38%
	>4 to 5	13	4.83	0.28	-1.99*	-41%	0.81	7	54%
	>5 to 6	16	5.97	0.12	-1.97*	-33%	0.80	10	63%
Severe	>6 to 7	9	6.82	0.26	-1.68*	-25%	0.84	3	33%
	>7	5	8.31	1.19	-3.72*	-45%	0.90	4	80%
	>1 to 10	110	4.15	1.83	-1.33*	-32%	0.66	41	37%

Therefore, for all classes, the hypothesis of no differences between the classes was rejected and the alternative hypothesis was accepted. Also, the second zero hypothesis “all classes improve significantly” was rejected and the hypothesis “not all classes improve significantly” was accepted.

## Discussion

The primary objective of this study was to investigate whether patients with different initial pain intensities derive varying degrees of benefit from WB-EMS treatment for nonspecific chronic back pain (NSCBP) and to ascertain if significant improvements are evident across all baseline pain intensity groups. The data suggests that individuals with higher baseline numeric rating scale (NRS) values experience greater reductions in pain following WB-EMS therapy. This finding is particularly salient for the cohort with initial pain scores exceeding 7, where the rate of clinically significant changes reached 80%, and the mean improvement was 3.72 points on the NRS. Such substantial improvements underscore the potential of WB-EMS as a highly effective intervention for patients suffering from severe NSCBP.

For individuals presenting with lower initial pain scores (0-2 on the NRS), the observed mean improvement was modest, at -0.14 points, indicating a slight reduction in pain. Although this group demonstrated the smallest mean improvement and the lowest rate of clinically significant improvements (4%), it is important to acknowledge the potential floor effect in this category, where individuals have less room for improvement due to already low baseline pain levels. This underscores the nuanced nature of pain management, where different strategies may be more or less effective based on initial pain severity.

In the intermediate pain categories, such as those with initial pain scores ranging from >2 to 6, we observed a gradation of improvements, with mean improvements ranging from -0.81 to -1.97 points and rates of clinically significant improvements increasing from 26% to 63% as initial pain scores increased. These categories reflect a substantial portion of the NSCBP population, highlighting WB-EMS's versatility as a therapeutic option capable of providing meaningful benefits across a broad spectrum of pain intensities.

Particularly noteworthy is the moderate pain category (>3 to 4), where a mean improvement of -1.34 points was observed, and 38% of the subjects improved clinically significantly. This category, along with the severe pain category (>5 to 6), illustrates the therapy's potential to effect significant pain reduction in those with moderate to severe NSCBP.

The analysis further revealed a moderate positive correlation between initial pain levels and the degree of pain reduction, suggesting a general trend that higher initial pain levels are associated with greater pain relief. This correlation is critical for understanding the differential efficacy of WB-EMS across various pain intensities and provides a robust basis for tailoring treatment to individual patient needs.

Interestingly, the study's findings challenge some prevailing assumptions about the effectiveness of different treatments for NSCBP. For instance, previous research by Berglund et al. [39] suggested that patients with high pain intensity do not benefit from certain rehabilitative exercises, such as deadlifts. In contrast, our results indicate that WB-EMS, which does not rely on external loading, can be particularly beneficial for this patient group. These results align with clinical expectations that patients with higher initial pain levels may experience more notable reductions post-treatment, potentially due to a higher ceiling for perceivable improvement.

This distinction highlights the unique advantages of WB-EMS, especially its ability to stimulate muscle groups without imposing additional strain on the back, making it a suitable option for individuals with high baseline pain levels.

The demographic composition of the study sample, with a predominance of female participants, mirrors the epidemiological distribution of NSCBP, which is known to affect females more frequently than males [4]. Despite the observed gender differences in the prevalence of NSCBP, our analysis found no significant gender-based differences in pain reduction outcomes. Numerous studies evaluate sex in the context of pharmacological pain treatments, while for non-pharmacological interventions, only a few studies are present [36]. In the review mentioned before, Fillingim et al. [36] showed that results from non-pharmacological interventions are inconsistent; however, a slight majority of analyzed studies suggest that gender does not have a significant impact in the context of non-pharmacological interventions and back pain.

While this alignment with known gender disparities in NSCBP incidence provides an accurate representation of the population most affected, it also raises considerations regarding the generalizability of our findings. Specifically, given that gender differences in response to various treatments for NSCBP have been documented, albeit inconsistently, the efficacy of WB-EMS in our predominantly female sample may not directly translate to male patients with NSCBP. Also, our data do not show gender-based differences in pain reduction; this aspect warrants further investigation to understand fully the implications of gender on treatment outcomes with WB-EMS and to ensure that our findings are applicable across the broader NSCBP patient population.

Regarding age, our findings align with existing data that suggests that older patients may have a poorer prognosis [35,38], albeit our results showed this trend without statistical significance.

Given the lack of significant findings regarding the influence of gender and age on NRS changes, it is inferred that these factors do not pose significant confounding effects in our analysis. The absence of significant distortion by age and gender suggests that, within the context of our study's non-pharmacological treatment approach, these demographic variables may not critically impact pain prognosis.

This re-analysis serves to deepen our understanding of the treatment of NSCBP with WB-EMS training. The findings elucidate which patients are most likely to benefit from WB-EMS therapy, thereby providing medical professionals with a crucial basis for treatment allocation.

While our study provides valuable insights into the pain level-dependent efficacy of WB-EMS for NSCBP, it is crucial to acknowledge the limitations inherent in our methodology, particularly regarding the validation of NRS scores. The utilization of different methods by Weissenfels et al. [27] and Konrad et al. [28] to validate NRS scores, ranging from multiple measurement approaches to cross-referencing with other pain scales, introduces variability that may affect the comparability and interpretation of results. The variability in validation approaches could lead to differing conclusions about the magnitude of pain reduction achieved, especially when assessing the threshold for clinically significant changes in pain intensity. Such variability underscores the need for standardized assessment tools in future research to enhance the validity and reliability of findings. Furthermore, it highlights the importance of cautious interpretation of our results within the context of these methodological differences and their potential impact on the study's overall validity.

## Conclusions

The data of this study indicate that the initial pain severity of NSCBP patients is a predictive factor for pain reduction by WB-EMS therapy, particularly highlighting the significant benefits for patients with higher initial pain intensities. Accordingly, WB-EMS may be recommended as a valuable alternative (or adjunct) to conventional treatment modalities for NSCBP, especially for those whose conditions are characterized by more severe pain levels. This recommendation may be predicated on the correlation observed between higher baseline pain intensities and the degree of pain reduction following WB-EMS therapy, which suggests a targeted potential for improving patient outcomes in more severe cases. Recognizing the trend of our findings, we advocate for the inclusion of WB-EMS in NSCBP treatment protocols, with a particular focus on its application for patients experiencing mid to high levels of baseline pain.

Despite the promising nature of these results, we acknowledge the necessity of further more generalizable research to corroborate and reinforce the findings of this study. Future studies should not only aim to include a larger number of participants to strengthen the statistical power and generalizability of the findings but should also explore additional research avenues. These include conducting long-term follow-ups to assess the sustainability of pain reduction, comparing WB-EMS with other established and emerging treatments to contextualize its effectiveness, and delving into the impact of demographic factors, such as age, gender, and socioeconomic status, on treatment outcomes. Such investigations will be crucial for developing a comprehensive understanding of WB-EMS's role in NSCBP management and for optimizing its implementation to maximize patient benefits. The call for a multifaceted approach to future research reflects our commitment to advancing the field of pain management and to addressing the complex needs of individuals suffering from NSCBP. By broadening the scope of investigation, we can further refine treatment strategies, ensuring they are both evidence-based and tailored to the diverse experiences of NSCBP patients.
